# Dyslexics’ Fragile Oculomotor Control Is Further Destabilized by Increased Text Difficulty

**DOI:** 10.3390/brainsci11080990

**Published:** 2021-07-27

**Authors:** Lindsey M. Ward, Zoi Kapoula

**Affiliations:** IRIS Lab, Neurophysiology of Binocular Motor Control and Vision, CNRS UAR 2022 Neurosciences, UFR Biomedical, University of Paris, 45 Rue des Saints Pères, 75006 Paris, France; lward@mednet.ucla.edu

**Keywords:** dyslexia, reading, oculomotor system, saccades, vergence

## Abstract

Dyslexic adolescents demonstrate deficits in word decoding, recognition, and oculomotor coordination as compared to healthy controls. Our lab recently showed intrinsic deficits in large saccades and vergence movements with a Remobi device independent from reading. This shed new light on the field of dyslexia, as it has been debated in the literature whether the deficits in eye movements are a cause or consequence of reading difficulty. The present study investigates how these oculomotor problems are compensated for or aggravated by text difficulty. A total of 46 dyslexic and 41 non-dyslexic adolescents’ eye movements were analyzed while reading *L’Alouette*, a dyslexia screening test, and *35 Kilos D’Espoir*, a children’s book with a reading age of 10 years. While reading the more difficult text, dyslexics made more mistakes, read slower, and made more regressive saccades; moreover, they made smaller amplitude saccades with abnormal velocity profiles (e.g., higher peak velocity but lower average velocity) and significantly higher saccade disconjugacy. While reading the simpler text, these differences persisted; however, the difference in saccade disconjugacy, although present, was no longer significant, nor was there a significant difference in the percentage of regressive saccades. We propose that intrinsic eye movement abnormalities in dyslexics such as saccade disconjugacy, abnormal velocity profiles, and cognitively associated regressive saccades can be particularly exacerbated if the reading text relies heavily on word decoding to extract meaning; increased number of regressive saccades are a manifestation of reading difficulty and not a problem of eye movement per se. These interpretations are in line with the motor theory of visual attention and our previous research describing the relationship between binocular motor control, attention, and cognition that exists outside of the field of dyslexia.

## 1. Introduction

Reading depends on multiple different processes: sensory perception, eye movements, and linguistic and semantic decoding [1]. Eye movements, or, more specifically, good control of the oculomotor system via saccades (to the next word), fixation (on the word), and vergence movements (used to adjust the angle of axes according to depth, enabling placement of the word on the two foveas), have been shown to be essential for efficient reading [2].

There have been multiple studies that have demonstrated deficits in eye movements in dyslexic children as compared to non-dyslexic children during a reading task [3,4,5]. It was previously found that dyslexics demonstrate an increased number of vergence errors during reading, thought to be secondary to deficits in binocular yoking of saccades [6]. Coordinated saccades are particularly important during reading in order to allow the brain single vision of letters and words. It is argued that the capacity to make coordinated saccades relies on adaptive mechanisms involving the interaction between vergence and saccades [3,6,7]. It has also been previously shown that, when mismatched vergence-accommodation is induced experimentally with the use of prisms or spherical lenses, reading saccades become more disconjugate, leading to residual disparities during subsequent fixation [8]. Indeed, in another particularly interesting study, binocular coordination and reading scores were studied in a group of students with demonstrated vergence problems. Once these vergence deficits were corrected, there was a decrease in the number of regressive saccades and fixation duration while reading [9].

Though many studies have demonstrated abnormal eye movements in the dyslexic population while reading, it is a matter of debate whether these abnormal oculomotor profiles contribute to poor reading skills or if they are the consequence of other deficits that impair reading performance. Usually, dyslexia is classified as a primary learning disability. It is believed that the primary deficit consists of difficulties with word recognition, decoding words, and spelling, which manifests itself as decreased reading speed, decreased comprehension, and trouble with writing [5,10]. Therefore, dyslexia is typically described as a learning disability that manifests itself at the behavioral level. One way to explain abnormal oculomotor movements in dyslexics is the magnocellular theory, which posits that dyslexics’ oculomotor abnormalities are associated with deficient visual perception that is causal for dyslexia [11]. Magnocells control the speed of the eye on the movement of the target, effectively controlling smooth pursuit of an object. They also moderate fixation in that they modulate eye muscle control to recenter the eye on a target. It is also thought that saccades are controlled via this pathway in that they detect the appearance of the target and its positioning in space. All this is to say, that it is believed that the magnocellular pathway is related to fixation stability, smooth pursuit, and saccades, and impairment of the pathway produces deficits in those parameters.

To support the magnocellular theory, there have been many studies which have demonstrated abnormal eye movements in the dyslexic population independent of reading. Many studies have shown deficits in the dyslexic population during eye movements to random targets that stimulate vergence and saccades. [6,7,12,13]. Dyslexics have been found to have deficiencies in fixation stability, poor smooth pursuit, and poor vergence amplitudes [14]. Other experiments have demonstrated increased latencies of vergence and saccadic movements [12,15,16,17]. Additionally, it has been found that dyslexics have trouble with binocular coordination during saccades and vergence movements independent of reading [6,13].

Though it has been demonstrated that dyslexics preserve these abnormal movements independent of reading, there has been debate over this theory, with researchers positing alternatively that the oculomotor behavior occurs as a consequence of difficulty in processing that dyslexics experience while reading [18,19]. Indeed, many researchers have attempted to reproduce experiments that produce eye movements that are similar to reading without undergoing the act of reading itself, with various results. Pavlidis performed a well-known early study in which dyslexics were asked to fixate on sequential dots that simulated the motion of reading without using words. Dyslexics showed that they tended to regress backwards to the previous dot more than non-dyslexics. This randomly generated test neither provides sensical clues to help the viewer remain engaged and moving from left to right nor provides words that necessitate decoding, yet dyslexics still have difficulty moving forward due to their poor oculomotor coordination [20]. Despite this evidence, since this paper was published there have been many studies that have produced inconsistent reproductions of the study, with some finding abnormal eye movements during a sequential processing task and others finding no significant difference [21,22,23,24,25,26].

Interestingly, Pollatsek proposed that the inability to replicate Pavlidis’ original study may be due to his subject selection, which he writes was biased towards those with a propensity for oculomotor problems [27]. Indeed, other research has demonstrated that dyslexics’ oculomotor deficits can improve with oculomotor training, which can also result in improved reading performance [9,28,29,30]. Other research has demonstrated that dyslexics oculomotor control develops with age, suggesting there is a training component to oculomotor control that could result in heterogeneity of subjects across different studies which could produce different results in confirming Pavlidis’ study [31].

Although differences in subjects could certainly produce differing results in the established literature, is there any role for how attention and engagement with the text, or how text choice itself, could affect eye movements? Some have argued that attention is a byproduct of eye movements [32,33,34,35,36]. Daniel et al. argue that the vector of visual attention lies in the interaction of saccade, vergence, and accommodation [8,9,35]. Fragile control of binocular eye movements could affect attention; reciprocally, a test that requires increased attention could destabilize an already fragile binocular motor control system. This phenomenon is akin to what patients experience during the cover-uncover test, which provokes heterophoria, or the loss of binocular alignment, when one eye is temporarily covered, interrupting binocular visual stimulation. Importantly, this test reveals heterophoria only in people with a weak binocular system. Similarly, loss or absence of sense in the reading text could affect binocular motor control in those with an already fragile binocular system. As there is a tight relationship between attention and eye movement control, we hypothesize this interaction could be bidirectional: could word decoding difficulty further deteriorate intrinsic eye movement deficits?

Given their abnormal oculomotor control and their difficulty decoding words, would a text with meaning and context clues that provide a more complicated engagement actually assist dyslexics with reading, as evidenced by decreased number of regressions? Or would a text that relies primarily on decoding words without context clues cause them to become less engaged and regress more often? Although eye movements, independent of, and during reading, have been shown to be abnormal in dyslexic adolescents, there is a large field of research demonstrating a cognitive learning disability centered in a problem with decoding words. Is it possible that there could be a mixed picture of deficits, in which oculomotor control is especially perturbed by a decreased ability to decode meaning in a particular text?

Therefore, we compared eye movements while reading two different texts in a dyslexic and non-dyslexic population. It has been shown that a text that provides context clues to make sense of the story is read faster than a text with words placed in random order [37]. Screening of dyslexia, therefore, often uses a text that creates normal reading conditions with a grammatically and syntactically correct text. However, the text lacks any sort of meaning, making it impossible to predict the content of the story moving forward in the text. Therefore, there are no clues the reader could use to compensate for inherent decoding difficulties that might be present in a reader [38]. In France, the “Alouette Test” is the most commonly used text to diagnose children and adults with dyslexia on the bases of abnormal scores of accuracy, speed, and efficiency and has been validated as a test with a high specificity and sensitivity for screening for dyslexia [39]. We compared eye movements and reading scores while dyslexics and non-dyslexics read this text and an engaging, sensical text extracted from a book targeted towards children of 10 years of age [40].

We propose that oculomotor deficits could be altered by the different characteristics of the reading text. A text which requires more decoding skills could exacerbate the fragility of eye movements; while a text that provides more context and predictability may not perturb the eye as much. More specifically, the question remains: which parameters are particularly modulated by the choice of text? Is it reading speed and regressive saccades, or parameters more specifically associated with motor control per se, such as saccade velocity or disconjugacy?

## 2. Materials and Methods

### 2.1. Participants

A total of 47 dyslexic adolescents (18 female, 29 male; mean age 15.4) and 44 non-dyslexic adolescents (22 female, 22 male; mean age 14.8) were selected from middle and high schools in Paris. Each dyslexic adolescent was given a diagnosis of dyslexia via extensive multidisciplinary testing in specialized centers (including neurological/psychological testing, evaluation of reading, comprehension, and capacity of reading words and pseudowords). Each dyslexic child was admitted to their school on the basis of their diagnosis. In breaking down the types of dyslexia in the population, based on school records, 34.0% (16/47) identified their primary problem was visual/reading based, 4.3% (2/47) was auditory, 2.1% (1/47) was writing, and 59.6 (28/47) were mixed or unknown. As is common in the dyslexic population, many had co-morbid conditions: twelve were diagnosed with dysorthographia, dyscalcula, and/or dyspraxia. As is common in France, 34 had been to an orthoptist or were currently enrolled in orthoptic rehabilitation. All participants had no known neurologic or psychiatric abnormalities. Non-dyslexic adolescents had no difficulty with vision, visual impairment, or difficulty reading. The investigation adhered to the principles of the Declaration of Helsinki and was approved by our Institutional Human Experimentation Committee (CPP CNRS 18 011). Written, informed consent was obtained from the adolescents and/or their parents after they were given an explanation about the experimental procedure.

### 2.2. Eye Movement Recording Device

Binocular eye movements were recorded at 200 Hz per eye with a head-mounted video-oculography device called Pupil Core (Pupil Labs, Berlin, Germany).

### 2.3. Calibration of the Recording Device

The device was calibrated with the standard calibration software (Pupil Capture, Pupil Labs). The subject fixated on the center of a target presented at a viewing distance of 1 m. They then moved their head at their own pace rightward, downward, leftward, and upward twice.

### 2.4. Texts

All children performed two visual reading tasks while seated comfortably at 40 cm viewing distance from a screen. Each adolescent was instructed to read the text out loud. First, each adolescent viewed binocularly the text *L’Alouette*, in 16 lines on black lines on a white background. *L’Alouette* is a text commonly used for evaluation of reading capacity in dyslexia, as the order of the words is unusual and contains uncommon words for children [39]. The second text was an excerpt of 15 lines in black text on a white background from a children’s book (*35 Kilos D’Espoir*, Anna Gavalda, Bayard Jeunesse) targeted towards children with a reading age of 10 years [40]. Please see Appendix A and Appendix B for examples of the text.

### 2.5. Data Analysis

The recorded data were analyzed with a software developed in the IRIS Laboratory called AIDEAL. The software treated the conjugate gaze signal, or the L + R eye position/2. The saccade was defined as the time at which the peak velocity was greater or less than 10% of the peak velocity; practically this meant that saccades were above or below 40°/s (as the peak velocity of 20° saccades is typically above 40°/s). Total average velocity was defined as total amplitude divided by time. Disconjugacy during saccadic movements, or the binocular coordination of saccades, was measured by the difference in amplitude between the left and the right eye signal. The difference in drift amplitude during the first 80 or 160 ms of fixation was calculated as the disconjugate drift. These calculations are standard and have been used in previous experiments [29,30].

Trials with blinks or other artifacts were discarded automatically by AIDEAL. For each adolescent (dyslexic and non-dyslexic) the number of saccades movements measured in the fixation tasks were counted. The percentage of movements rejected was 1.05% for dyslexic adolescents and 2.27% in healthy adolescents.

### 2.6. Statistical Analysis

As the measured eye movements data were not normally distributed as determined by the Shapiro–Wilk test, the non-parametric Mann–Whitney U test was utilized for means comparison. In a first analysis, we compared parameters between the dyslexic and non-dyslexic populations while reading *L’Alouette*. In a second test, we compared parameters between the two populations while reading an excerpt from *35 Kilos D’Espoir*. We then compared parameters between reading Text 1 vs. Text 2 for the entire population. In a fourth test, we compared parameters between reading Text 1 vs. Text 2 in the dyslexic population only. Finally, we compared parameters between reading Text 1 and Text 2 in the healthy control population only. We did not attempt to correct for multiple comparisons.

For all analyses, the statistical significance was set at *p* ≤ 0.05. All analyses were performed using SPSS version 25 (IBM Corp. Released 2017. IBM SPSS Statistics for Windows, Version 25.0. IBM Corp. Armonk, NY, USA).

## 3. Results

### 3.1. Comparing Reading Performance and Eye Movements in each Text by Population

In comparing dyslexic adolescents to healthy controls while reading Text One (*L’Alouette*), dyslexics exhibited several abnormalities in eye movement control as compared to non-dyslexics: a smaller amplitude (1.93°, SD 0.36 vs. 2.23°, SD 0.45; *p* < 0.001), a longer duration (64.97 ms vs. 44.07 ms, *p* = 0.001), a higher peak velocity (80.82 °/ms vs. 66.30 °/ms; *p* = 0.005) yet a lower average velocity (42.13°/ms vs. 64.24°/ms, *p* < 0.001), and a larger fixation disconjugacy during the saccade (0.76° vs. 0.61°, *p*–0.037). These findings are consistent with eye movement abnormalities previously found in dyslexics independent of reading using Remobi testing [13]. They also had a larger percentage of regressive saccades (saccades to the left) (35% vs. 28%; *p* = 0.014), made more mistakes per word of text (0.06 mistakes/word vs. 0.03 mistakes/word, *p* < 0.001), and read more slowly (92 words per minute vs. 136 words per minute; *p* < 0.001) (See Table 1).

In comparing the two populations while reading the simpler text, these differences persisted though there were a few notable exceptions. There was no longer any significant difference between the populations in the fixation disconjugacy during the saccade, nor in the percentage of regressive saccades (See Table 2).

### 3.2. Comparing Texts in Each Population

There were also significant differences in the dyslexic population in reading the two texts (see Table 3). When reading *L’Alouette*, dyslexics displayed a smaller amplitude (1.93° vs. 2.15°; *p* = 0.004) yet a faster velocity (42.13°/ms vs. 52.92°/ms; *p* = 0.007), as compared to when they were reading the less complicated text, *35 Kilos D’Espoir*. This may be expected given one would expect a smaller saccade would necessitate a shorter velocity to make the movement. They also made more regressive saccades (35% vs. 31%, *p* = 0.036), made more mistakes per word of text (0.06 vs. 0.03, *p* < 0.001), and read more slowly (92 words per minute vs. 124 words per minute, *p* < 0.001).

Healthy controls, unsurprisingly, also displayed differences in eye movements and reading behavior between the two texts (see Table 4). When reading *L’Alouette*, healthy controls also displayed a smaller amplitude (2.23° vs. 2.56°; *p* = 0.002), made more mistakes per word (0.026 vs. 0.015, *p* < 0.001), and read more slowly (136 words per minute vs. 176 words per minute, *p* < 0.001). The majority of these differences between reading texts independent of population tested are related to language processing (regressive saccades, mistakes per word, and word per minute) and not to eye movement control per se, reflecting the cognitive aspects of oculomotor control.

## 4. Discussion

### 4.1. Velocity Profile during Reading Demonstrate Poor Oculomotor Coordination

Similar to our previous study of dyslexic and non-dyslexic vergence and saccade movements to randomly generated targets, we found that dyslexics’ reading saccades also showed abnormal velocity profiles [13]. As peak velocity, which is achieved early in the trajectory of the movement, is higher in dyslexics, a slower average velocity indicates slowing of the deceleration in the subsequent phase of movement. We attributed this slowing to poor control of vergence during the saccade that is necessary to keep binocular eye alignment during and after the movement.

There is one important difference in eye movement parameters between the two texts. While reading *L’Alouette*, the text that required greater use of word decoding skills, dyslexics demonstrated a greater disconjugacy during the saccade, meaning that their eyes were poorly coordinated as they moved from one word to the next. This finding is similar to our previous study using non-reading conditions, which showed a greater disconjugacy of saccades to audiovisual targets in dyslexics.

In the present study, abnormal disconjugacy appeared once again, though only while reading *L’Alouette*. When adolescents are confronted with a text that relies heavily on word decoding and provides no context clues to help extract meaning, perhaps the binocular motor coordination system, which hypothetically involves saccade-vergence interplay, is less efficient, thereby leading to disconjugacy. Reading normally implies a rather automatic motor sequence of the eyes from left to right. Dyslexics’ difficulty in decoding words to extract meaning exacerbates dyslexics’ already fragile oculomotor system, not only potentially revealing abnormal velocity profiles and greater disconjugacy during eye movements, but also creating more regressions, more mistakes, and a slower reading time. In other words, we propose that lack of sense destabilizes both motor aspects and cognitive aspects of eye movement control in dyslexia.

Interestingly, dyslexic children were shown to coordinate their eyes just as well as non-dyslexic children while reading *35 Kilos D’Espoir*, the text which provides greater context clues to the reader and decreases reliance on word decoding ability. Perhaps when dyslexics read a text that carries more context clues and does not rely heavily on word decoding skills, they are able to extract meaning and move more smoothly along a line of text similarly to non-dyslexic readers. These observations are in line with our previous studies [8,9]. Given that these eye movement abnormalities in saccades and vergence exist outside of reading, we propose that the difference between the two reading texts presented here could be understood as evidence for a fragile oculomotor system that is perturbed when attempting to decode a meaningless text.

### 4.2. Regressive Saccades Provide Insight into How Dyslexics Internalize Reading Text

There are some new insights we can gain into the differences between dyslexic and non-dyslexic eye movements during reading. When reading *L’Alouette*, the text which requires the reader to rely more heavily on individual word decoding skills, dyslexics exhibited more regressive saccades as compared to non-dyslexics, meaning their eyes moved backwards to the previous word more frequently, indicating difficulty with fully internalizing the word. Though we would expect this to be true of how dyslexics read all texts, we found that there was actually no significant difference in regressive saccades between the populations while reading *35 Kilos D’Espoir*, a text that provided more context clues and narrative to create a cohesive story.

It is important to note that these regressive saccades more likely represent a cognitive disability as opposed to a pure oculomotor deficit. As dyslexics had more difficulty during word decoding, they attempted to regress backwards to the previous word in order to search for a coherent story to find meaning in the text. Non-dyslexics, who do not struggle with word decoding, are more likely to understand the meaning of the word as they progress across the line and do not have to search for context clues via regressive saccades. It is notable that dyslexics did not have more regressive saccades as compared to non-dyslexics when reading *35 Kilos D’Espoir*. We postulate this is because the latter text provides more context clues throughout the text to construct a coherent story, so that dyslexics can understand the trajectory of the narrative without needing to focus as much on the meaning of each word. This concept is confirmed in the fact that dyslexics also showed more regressive saccades when reading *L’Alouette* as compared to reading *35 Kilos D’Espoir*. Even though dyslexics have a fragile oculomotor system with poor coordination, they demonstrated more regressive saccades in the text with higher word decoding requirements, confirming that these regressive saccades are more likely secondary to a cognitive issue than a motor one.

Regressive saccades can represent a healthy phenomenon in that it could be the result of a trigger to move backwards when one views a word that is incomprehensible. Therefore, these regressive saccades are not purely or necessarily triggered by pure intrinsic motor control, and it is likely that a portion of these regressive saccades are related to a poor cognitive recognition of the material.

### 4.3. Cognitive Aspects of Reading Differences

From a cognitive perspective, dyslexics exhibit increased errors and a slower reading speed in both texts, confirming dyslexics do indeed read more slowly and with more difficulty, regardless of the text or its cognitive load.

Additionally, there were significant differences in amplitude in each of the comparison groups. Dyslexics demonstrated a smaller amplitude as compared to non-dyslexics while reading both the difficult and the easier text. Previous studies have demonstrated different reading strategies in the dyslexic vs. non-dyslexic population, in which dyslexics preferentially utilize an indirect grapheme/phoneme strategy, breaking words down into smaller pieces and therefore using a smaller amplitude to move across the word in shorter segments. Alternatively, non-dyslexics favor a whole word lexical strategy, which would provide a larger amplitude as the adolescent moves across the whole word [41]. Werth has also found that segmentation of text and longer fixation times improves oculomotor abnormalities [42,43]. It is possible that segmenting the text into smaller portions reduces the need for accommodative effort, and therefore for accommodative vergence, decreasing the stress on the dyslexic child and allowing for more coordinated movements. This is, unfortunately, not sustainable for dyslexic readers and ultimately not representative of how readers view the world around them; by changing the way the subject sees the text, they also change the way the visual system absorbs that text. This demonstrates a more integrative view of how various sensory inputs can influence oculomotor abnormalities and the general presentation of dyslexia; giving a more nuanced picture of the etiology of the condition.

When comparing the texts within each population, both dyslexics and non-dyslexics demonstrated smaller amplitudes while reading the more difficult text. Dyslexics and non-dyslexics both had a larger amplitude while reading the second text, which was easier. Perhaps the easier text has words that dyslexics are able to understand with an easier cognitive load, allowing them to internalize the word as a whole instead of breaking it down into smaller portions.

This modulation in amplitude demonstrates that there may be oculomotor parameters that could be a reflection of alternative cognitive perception in the dyslexic population. Therefore, it appears that one could consider a multidimensional approach to dyslexia, in which oculomotor control and cognition are closely related.

### 4.4. Limitations

As mentioned in the introduction, it has been shown that dyslexic oculomotor control can be improved with training exercises and generally improves with age. Many of our dyslexic subjects have participated in orthoptic reeducation, which is common in France. Indeed, our subjects were selected from a school that specializes in educating dyslexics, with specific classes for reading acquisition. Children in these schools are closely monitored at all levels (many years of speech, visual, and/or orthoptic therapy etc.). However, despite their enrollment in these programs, our findings still demonstrated significant differences in their oculomotor profile and reading ability, suggesting that neither speech therapy nor the standard of care orthoptic reeducation are adequately targeted towards improving oculomotor movements and reading skills.

## 5. Conclusions

This study shows that there are both cognitive and oculomotor components to the differences in how dyslexics read compared to their healthy peers. A text can induce word decoding difficulties in its structure that may worsen dyslexics already fragile oculomotor system. From this study, the *L’Alouette* test, which has been widely used throughout France in screening for dyslexia, proves to be a valid test to tease out not only decreased reading speeds and increased mistakes, but also a fragile oculomotor system in a dyslexic population. In addition to simply counting mistakes and measuring reading time, clinicians can use the velocity profile of saccade and vergence eye movements during and independent of reading in conjunction with the standard dyslexia tests, to measure eye movement fragility. As the *L’Alouette* test is considered to be predictive of dyslexia, perhaps there could a larger investigation to determine if it is the text or the eye movement abnormality itself caused by such a text that enables differences predictive of dyslexia. Future research should also be conducted in other languages and countries using text analysis and oculomotor monitoring to further understand the relationship between word decoding difficulties and oculomotor deficits in the dyslexic population.

## 6. Patents

Zoi Kapoula has applied for patents for the technology used to conduct this experiment: REMOBI table (patent US8851669, WO2011073288); AIDEAL analysis software (EP20306166.8, 7 October 2020; EP20306164.3, 7 October 2020–Europe).

## Figures and Tables

**Table 1:** Reading *L’Alouette*.

	Dyslexic		Non-Dyslexic		*p*-Value
	Median	SD	Median	SD	
Amplitude (deg)	1.93	0.36	2.23	0.45	<0.001
Duration (ms)	64.97	67.99	44.07	48.70	<0.001
Peak Velocity (deg/s)	80.82	35.49	66.30	41.68	0.01
Average Velocity (deg/s)	42.13	16.14	64.24	19.81	<0.001
Fixation Disconjugacy 80 msec after Saccade (deg)	0.36	0.37	0.037	0.43	0.92
Fixation Disconjugacy 160 msec after Saccade (deg)	0.61	0.64	0.56	0.60	0.64
Disconjugacy During Saccade (deg)	0.76	2.06	0.61	1.01	0.04
Fixation Duration (ms)	410.28	88.08	447.58	91.49	0.05
Percent Regressive Saccades	35.46		28.24		0.01
Mistakes per Word	0.06	0.06	0.026	0.02	<0.001
Words per Minute	92	109	136	29	<0.001

**Table 2:** Reading 35 *Kilos D’Espoir*.

	Dyslexic		Non-Dyslexic		*p*-Value
	Median	SD	Median	SD	
Amplitude (deg)	2.15	0.50	2.56	0.44	0.00
Duration (ms)	55.71	21.97	40.54	111.66	0.04
Peak Velocity (deg/s)	77.77	18.74	69.92	38.63	0.04
Average Velocity (deg/s)	52.92	17.19	76.56	21.57	0.00
Fixation Disconjugacy 80 msec after Saccade (deg)	0.32	0.19	0.37	0.27	0.28
Fixation Disconjugacy 160 msec after Saccade (deg)	0.50	0.36	0.54	0.50	0.72
Disconjugacy During Saccade (deg)	0.64	1.84	0.64	0.96	0.55
Fixation Duration (ms)	381.66	54.99	406.15	93.06	0.10
Percent Regressive Saccades	31.44		26.56		0.42
Mistakes per Word	0.03	0.03	0.015	0.01	0.00
Words per Minute	124	33	176	34	0.00

**Table 3:** Comparing Texts in the Dyslexic Population.

	Text 1		Text 2		*p*-Value
	Median	SD	Median	SD	
Amplitude (deg)	1.93	0.36	2.15	0.50	0.00
Duration (ms)	64.97	67.99	55.71	21.97	0.07
Peak Velocity (deg/s)	80.82	35.49	77.77	18.74	0.22
Average Velocity (deg/s)	42.13	16.14	52.92	17.19	0.01
Fixation Disconjugacy 80 msec after Saccade (deg)	0.36	0.37	0.32	0.19	0.33
Fixation Disconjugacy 160 msec after Saccade (deg)	0.61	0.64	0.50	0.36	0.29
Disconjugacy During Saccade (deg)	0.76	2.06	0.64	1.84	0.11
Fixation Duration (ms)	410.28	88.08	381.66	54.99	0.25
Percent Regressive Saccades	35.46		31.44		0.04
Mistakes per Word	0.06	0.06	0.03	0.03	0.00
Words per Minute	92	109	124	33	0.00

**Table 4:** Comparing Texts in the Control Population.

	Text 1		Text 2		*p*-Value
	Median	SD	Median	SD	
Amplitude (deg)	2.23	0.45	2.56	0.44	0.00
Duration (ms)	44.07	48.70	40.54	111.66	0.70
Peak Velocity (deg/s)	66.30	41.68	69.92	38.63	0.76
Average Velocity (deg/s)	64.24	19.81	76.56	21.57	0.06
Fixation Disconjugacy 80 msec after Saccade (deg)	0.037	0.43	0.37	0.27	0.97
Fixation Disconjugacy 160 msec after Saccade (deg)	0.56	0.60	0.54	0.50	0.61
Disconjugacy During Saccade (deg)	0.61	1.01	0.64	0.96	0.82
Fixation Duration (ms)	447.58	91.49	406.15	93.06	0.22
Percent Regressive Saccades	28.24		26.56		0.91
Mistakes per Word	0.026	0.02	0.015	0.01	0.00
Words per Minute	136	29	176	34	0.00

## Data Availability

The datasets generated during and/or analyzed during the current study are available from the corresponding author on reasonable request.
